# Scale‐dependent effects of herbivory on moss communities in Arctic wetlands: A 25‐year experiment

**DOI:** 10.1002/ece3.11272

**Published:** 2024-04-25

**Authors:** Chao Liu, Gilles Gauthier, Charles Gignac, Esther Lévesque, Line Rochefort

**Affiliations:** ^1^ Centre d'études Nordiques Université Laval Québec Québec Canada; ^2^ Département de Phytologie Université Laval Québec Québec Canada; ^3^ Département de Biologie Université Laval Québec Québec Canada; ^4^ Laboratoire d'Écologie Végétale Fonctionnelle (LEAF), Département des Sciences de l'Environnement Université du Québec à Trois‐Rivières Trois‐Rivières Québec Canada

**Keywords:** biodiversity, exclosure, plant population and community dynamics, plant–herbivore interactions, polygon fen, snow goose

## Abstract

Arctic ecosystems are undergoing rapid changes, including increasing disturbance by herbivore populations, which can affect plant species coexistence and community assemblages. Although the significance of mosses in Arctic wetlands is well recognized, the long‐term influence of medium‐sized herbivores on the composition of moss communities has received limited attention. We used data from a long‐term (25 years) Greater Snow Goose (*Anser caerulescens atlanticus*) exclusion experiment in Arctic tundra wetlands to assess changes in the composition of moss communities at multiple spatial scales (cell, 4 cm^2^; quadrat, 100 cm^2^; exclosure, 16 m^2^). We investigated how snow goose grazing and grubbing can alter the composition of the moss community by measuring changes in alpha and beta diversity, as well as in the strength of plant interspecific interactions between moss species. Our results indicate that goose foraging significantly increased species diversity (richness, evenness, and inverse Simpson index) of moss communities at the cell and quadrat scales but not the exclosure scale. Goose foraging reduced the dissimilarity (beta diversity) of moss communities at all three scales, mainly due to decreased species turnover. Furthermore, goose foraging increased positive interaction between moss species pairs. These findings emphasize the critical role of geese in promoting moss species coexistence and increasing homogeneity in Arctic wetlands. This study illustrates how top‐down regulation by herbivores can alter plant communities in Arctic wetlands and highlights the importance of considering herbivores when examining the response of Arctic plant biodiversity to future climate change.

## Graphical abstract

Our study shows the critical role of goose herbivory in promoting the coexistence of moss species and increasing homogeneity. At intermediate levels of herbivory, goose foraging as a driver of spatial patterns in plant communities and may play an important role in the conservation of Arctic tundra wetlands in a context of global change.

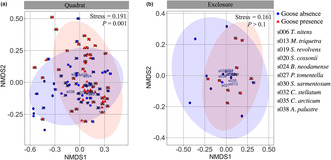

## 1 INTRODUCTION

Herbivores have widespread effects on the structure and diversity of plant communities in ecosystems worldwide (Alberti et al., 2017; Jia et al., 2018). Herbivory can regulate ecological systems in a “top‐down” manner by decreasing plant survival, biomass, and abundance, but it can also promote diversity (species richness and evenness) by selectively consuming dominant species, which prevents competitive exclusion (Jia et al., 2018; Veen et al., 2008). Additionally, herbivory can alter the spatial heterogeneity of vegetation (beta diversity) by grazing selectively in patches or grazing homogeneously (Adler et al., 2001; Beguin et al., 2022). Understanding the impacts of herbivores on the biodiversity of plant communities is crucial for effective conservation.

Geese are common, medium‐size herbivores in the Arctic and many populations have increased dramatically worldwide, in large part due to food subsidy obtained in agricultural landscapes in the winter (Abraham et al., 2005; Fox & Madsen, 2017; Gauthier et al., 2005). Overabundant goose populations have greatly impacted tundra vegetation in several regions, leading to habitat destruction in some extreme cases (Jefferies et al., 2006; Speed et al., 2009). A prominent example is provided by the Greater Snow Goose (*Anser caerulescens atlanticus*, hereafter referred to as snow geese), a population that breeds in the Canadian High Arctic and which experienced a large population increase in the late 20th century (Gauthier et al., 2005). Snow geese primarily feed on above‐ground graminoids and can consume up to 60% of the annual production in Arctic wetlands during the summer (Gauthier et al., 1995; Valéry et al., 2010). Such intense grazing in plant communities characterized by low primary productivity can profoundly impact plant production and community composition (Deschamps et al., 2022; Gauthier et al., 2004; Nishizawa et al., 2021).

**FIGURE 1:**
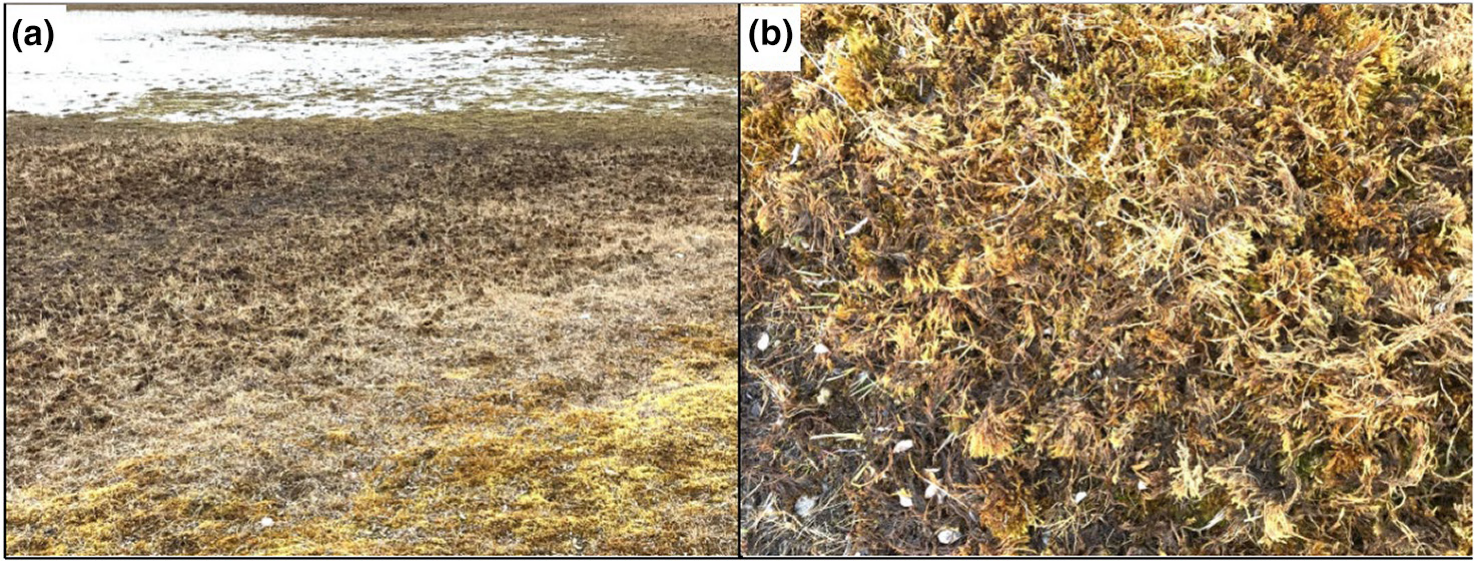
Effect of snow goose grubbing on vegetation structure on Bylot Island in the Canadian Arctic. Grubbing reduces standing biomass of graminoids and disrupts the moss layer.

Mosses constitute a significant component of peatland ecosystems, including in Arctic wetlands used by foraging snow geese (Gauthier et al., 1996, 2004). Mosses can structure plant diversity, contribute to carbon sequestration and nitrogen fixation, and account for a substantial proportion of net primary production in the Arctic (Jägerbrand et al., 2006; Rzepczynska et al., 2022; Turetsky et al., 2012). Although snow geese do not typically consume mosses (Audet et al., 2007; Gauthier, 1993), they disrupt the moss layer due to their digging behavior when they forage on graminoid rhizomes, a behavior known as grubbing (Jasmin et al., 2008). Goose grubbing is often patchily distributed due to the presence of snow and frozen ground in spring, and it can be a major disturbance to the moss carpet and its plant structure (Figure 1). This process provides greater establishment opportunities for mosses, which can promote species richness and coexistence (Gauthier et al., 2004; Jasmin et al., 2008). However, previous studies examining these questions were relatively short‐term (<12 years) and no study has looked at the effects of snow geese on moss community dissimilarity among patches (beta diversity) at multiple scales.

Herbivores could alter competitive interactions among species, thus facilitating plant coexistence (Kempel et al., 2015; Olofsson et al., 2002; Pacala & Crawley, 1992; Souza et al., 2022). The mechanisms through which herbivores facilitate species coexistence include two main mechanisms: (i) Herbivores can suppress dominant species through disturbance due to feeding or grubbing, reducing the abundance of dominant species and promoting the predominance of subordinate species (Connell, 1971; Louda et al., 1990); (ii) herbivores can modify the environment by altering nutrient levels or reducing shading, creating conditions that promote the establishment or proliferation of certain species (Borer et al., 2014; Huntly, 1991). For instance, herbivores can decrease canopy cover and increase light availability for shorter plants by consuming vegetation, particularly when they target taller species that compete for light (Eskelinen et al., 2022). Therefore, by consuming vascular plants snow geese have the potential to alleviate moss competition for light and maintain diversity.

The ability of individual plants to regenerate dictates their response to disturbance and hence the composition of the community (Latzel et al., 2008; Sousa, 1980). Habitats subject to repeated disturbance are typically dominated by species that have a strong ability to regenerate (Benvenuti, 2004). Mosses can be broadly classified into two types based on their growth form: Acrocarpous, which grow sporophytes from the tips of their stems or branches, and Pleurocarpous, which develop sporophytes on their lateral branches. Generally, acrocarps grow more slowly than pleurocarps and do not regenerate from fragments as quickly as pleurocarps (Glime, 2017). As a result of this difference in regeneration ability, pleurocarps may be favored over acrocarps after goose grubbing.

Here we explore how snow goose foraging activity alters moss community composition via direct effects on alpha and beta diversity and indirectly by changing the strength of competitive interactions between moss species. To do so, we surveyed the abundance of individual moss species inside exclosures where snow geese had been excluded for a long time (25 years) and outside at three spatial scales (cm to m scales) in an Arctic wetland (Bylot Island in Nunavut, Canada). Our hypotheses are:

Goose foraging increases alpha diversity (species richness, evenness, and inverse Simpson index) of moss communities because they open regeneration niches when consuming graminoid rhizomes.

Goose foraging promotes the coexistence of moss species by enhancing positive associations.

Goose foraging decreases beta diversity (community dissimilarity) of moss communities by reducing species turnover because they may spread moss fragments to other areas during their foraging.

Pleurocarp growth forms are favored over acrocarp ones in areas disturbed by goose foraging, primarily because of their superior capacity to regenerate through vegetative reproduction.

## 2 MATERIALS AND METHODS

### 2.1 Study site

We conducted this study in tundra wetlands, mostly fens created by polygon‐patterned permafrost (Ellis et al., 2008), located in the Qarlikturvik glacial valley on Bylot Island in Nunavut, Canada (73°N, 80°W). This region is the most important breeding site of the greater snow goose in the Arctic. Each summer, a population of snow geese estimated at 25,000 pairs breeds in one large colony covering approximately 65 km^2^ on the south plain of Bylot Island (Reed et al., 1992). Snow goose foraging activities occur primarily in wetland habitats throughout the island during brood rearing (Masse et al., 2001). Importantly, due to the absence of large herbivorous mammals such as caribou (*Rangifer tarandus*) or muskoxen (*Ovibos moschatus*), the snow goose is the largest and most abundant herbivore on the island (Gauthier et al., 2011). The diet of snow geese on Bylot Island is mainly composed of graminoids, *Carex aquatilis* var. *stans* Drej., *Eriophorum scheuchzeri* Hoppe., and *Dupontia fisheri* R. Br. (Manseau & Gauthier, 1993). All these vascular plants are dominant species in the wetlands we studied.

Polygon fens are also covered by a thick layer of brown mosses disturbed by snow geese when they forage on graminoid rhizomes. The dominant mosses are *Scorpidium cossonii* (Schimp.) Hedenäs., *Scorpidium revolvens* (Sw.) Rubers., *Bryum neodamense* Itzigs., *Meesia triquetra* (L. ex Jolycl.) Ångström., *Campylium stellatum* (Hedw.) C.E.O. Jensen., *Sarmentypnum sarmentosum* (Wahlenb.) Tuom. & T.J. Kop., *Aulacomnium palustre* (Hedw.) Schwägr, and *Aulacomnium acuminatum* (Lindb. et Arnell) Kindb.

### 2.2 Experiment design and sampling

A total of 18 goose exclosures (4 m × 4 m) were randomly established in polygon fens of the study area in 1994 across a 3 km^2^ area (Gauthier et al., 2004). The exclosures were made of 2.5 cm mesh chicken wire, standing 50 cm tall and covered with a lightweight nylon netting (Gauthier et al., 2004). Exclosures were inspected and repaired annually to ensure that no geese could enter. The control plots were the nearby polygon fens outside the exclosure and, initially, these plots comprised vegetation similar to those inside the exclosures. In 2019, ten pairs of plots, each composed of one exclosure (only those that were still in good state and not influenced by major disturbances such as polar bears, landslides, or ice‐wedge degradation) and one adjacent control area were sampled.

We first positioned five quadrats (10 cm × 10 cm) inside each exclosure and five quadrats outside the exclosure over a similar‐size area in the same polygon fen. Quadrats were positioned according to the following criteria: (i) They were at a distance of at least 30 cm from the chicken‐wire fence and (ii) they were randomly thrown in areas with mosses, avoiding standing water or areas without mosses, which constituted <10% of the area (Jasmin et al., 2008). We harvested mosses on each quadrat by cutting to a depth of 10 cm. Each quadrat was then divided into 25 2 cm × 2 cm cells. Mosses present in each cell were dried in paper envelopes for 24 h at 50°C or until a constant weight was reached and brought back to the lab for analysis. In the laboratory, we identified each moss species and counted the total number of individual shoots of each species in each cell (total of 2500 cells), which was our measure of abundance. The design was thus hierarchically structured, with three nested levels: cells (4 cm^2^; *N* = 25/quadrat) within quadrats (100 cm^2^; *N* = 5/exclosure) within exclosures (16 m^2^; *N* = 10).

### 2.3 Measures of moss diversity

Measures of moss diversity used in this study included alpha diversity (species richness, evenness, and inverse Simpson index) and beta diversity (community dissimilarity, species turnover, and species nestedness).

Species richness, evenness, and inverse Simpson index were calculated at each spatial scale (cell, quadrat, and exclosure) for each treatment (presence or absence of snow geese). Species richness is the number of species recorded. Evenness (*E*) refers to how similar in numbers each species is in the environment. Evenness was calculated as follows (Camargo, 1993),

(1) $$\;E=1-2/\pi \;\arctan\;\left\{\sum_{i=1}^{S}{\left(\ln\left(x_{i}\right)-\sum_{j=1}^{S}\ln\left(x_{j}\right)/S\right)}^{2}/S\right\}$$

where *S* is the total number of species in a sampled plot and *x*_*i*_ and *x*_*j*_ is the abundance of the *i* ^th^ and *j* ^th^ species. It is based on the variance in abundance across species, which provides an intuitive measure of evenness (Smith & Wilson, 1996). The value ranges from 0 to 1, with 0 representing minimum evenness and 1 maximum.

The inverse Simpson index was preferred to other measures of alpha diversity because it is an indication of richness in a community with uniform evenness that would have the same level of diversity (Simpson, 1949). The inverse Simpson index (1/*λ*) was calculated as follows:

(2) $$\;\frac{1}{\;\lambda }=\frac{1}{\sum_{i=1}^{S}P_{i}^{2}}$$

(3) $$\;P_{i}^{2}=\frac{n_{i}\left(n_{i}-1\right)}{T\left(T-1\right)}$$

where *S* is the total number of species, *P*_*i*_ is the proportional abundance of *i* ^th^ species, *T* is the total number of individuals in the sampled plot, and *n*_*i*_ is the number of individuals of *i* ^th^ species in the sampled plot. A higher inverse Simpson index indicates greater diversity in the sample. In other words, the more evenly distributed the individuals are among different species in the sample, the higher the inverse Simpson index will be.

Beta diversity was estimated by community dissimilarity using Bray–Curtis dissimilarity metrics based on abundance data. This index is most suitable for non‐normal, multivariate data and is less affected by variations in rare species (Anderson & Walsh, 2013). The beta diversity (*β*_BC_) can be partitioned into components accounting for (i) balanced variation in abundance (turnover, *β*_bal_), whereby individuals of some species at one site are substituted by the same number of individuals of different species in another site and (ii) abundance gradients (nestedness, *β*_gra_), whereby some individuals are lost from one site to another (Baselga, 2016). The beta diversity (*β*_BC_), turnover (*β*_bal_), and nestedness (*β*_gra_) were calculated as follows:

(4) $$A=\sum_{i}\min\left(x_{\mathrm{ij}},x_{\mathrm{ik}}\right)$$

(5) $$B=\sum_{i}x_{\mathrm{ij}}-\min\left(x_{\mathrm{ij}},x_{\mathrm{ik}}\right)$$

(6) $$C=\sum_{i}x_{\mathrm{ik}}-\min\left(x_{\mathrm{ij}},x_{\mathrm{ik}}\right)$$

(7) $$\beta_{\mathrm{BC}}=\frac{B+C}{2A+B+C}=\beta_{\mathrm{bal}}+\beta_{\mathrm{gra}}$$

(8) $$\beta_{\mathrm{bal}}=\frac{\min\left(B+C\right)}{A+\min\left(B+C\right)}$$

(9) $$\beta_{\mathrm{gra}}=\frac{|B-C|}{2A+B+C}\times \frac{A}{A+\min\left(B+C\right)}$$

where *A* is the abundance of species that are both in plots *j* and *k*, *B*, and *C* are the abundance of unique species in plot *j* and plot *k*, respectively, *x*_*ij*_ is the abundance of species *i* in plot *j*, and *x*_*ik*_ is the abundance of species *i* in plot *k*. The *β*_BC_, *β*_bal_, and *β*_gra_ were also calculated at different scales (cell, quadrat, and exclosure) for each treatment (presence or absence of snow geese). All values range from 0 to 1. High values of *β*_BC_ indicate that communities of sampled plots are more dissimilar. High values of *β*_bal_ indicate that community dissimilarity is induced more by species replacement than by changes in total cover.

### 2.4 Species contribution to beta diversity

The Species contribution to beta diversity (SCBD) reflects the relative contribution of each species to the beta diversity pattern (Legendre & De Cáceres, 2013). SCBD was calculated as follows:

(10) $${\text{SCBD}}_{i}=\frac{\sum_{i=1}^{N}{\left(x_{\mathrm{ij}}-x_{i}\right)}^{2}}{\sum_{i=1}^{S}\sum_{j=1}^{N}{\left(x_{\mathrm{ij}}-x_{i}\right)}^{2}}$$

where *S* is the total number of species, *N* is the number of plots, *x*_*ij*_ is the abundance of specie *i* in plot *j*, and *x*_*i*_ is the mean abundance of specie *i* in all plots. The SCBD value ranges from 0 to 1. High SCBD values indicate that a species is an important contributor to beta diversity.

### 2.5 Interspecific association

The variance ratio (*VR*) test was used to gain insights into the overall interspecific association among the different species, and significance was tested using the *W* statistic value (Schluter, 1984). The formulas used are

(11) $$P_{i}=n_{i}/N$$

(12) $$\mathrm{VR}=S_{T}^{2}/\delta_{T}^{2}=\left[\frac{1}{N}\sum_{i=1}^{N}{\left(T_{j}-t\right)}^{2}\right]/\sum_{i=n}^{S}P_{i}{\left(1-P_{i}\right)}^{2}$$

(13) W=VR×N

where *n*_*i*_ is the number of plots containing species *i*, *N* is the total number of plots, *S* is the total number of species, *T*_*j*_ is the number of species occurring in plot *j*, and *t* is the average number of species in all plots. If *VR* > 1, species have a positive association, if *VR* < 1, species have a negative association, and if *VR* is close to 1 species have no association and they are considered independent. The overall interspecific association is significant (*p* < .05) when *W* < *χ* ^2^_0.95(*N*)_ or *W* > *χ* ^2^_0.05(*N*)_.

### 2.6 Statistical analyses

All analyses were performed in R v.4.0.2 (R Core Team, 2020). The overall relative frequency of each species was expressed as the ratio of the frequency of each species in all cells (*N* = 2500) to the sum of all species frequencies. We calculated the species richness, evenness, inverse Simpson index, beta diversity (*β*_BC_), turnover (*β*_bal_), nestedness (*β*_gra_) and species contribution to beta diversity (SCBD) using the R packages *BAT* (Cardoso et al., 2022), *Vegan* (Oksanen et al., 2019), *betapart* (Baselga et al., 2022), and *adespatial* (Dray et al., 2022).

First, we tested the influence of goose presence (fixed effect) on the abundance of each species or genus, as well as each measure of the diversity of the moss community at the cell, quadrat and exclosure scales, using a generalized linear mixed model (GLMM) with the function *glmer* from the R package *lme4* (Bates et al., 2015). In the model, the paired exclosure and adjacent control plot (hereafter referred to as Pairs) was the random variable. At the cell scale, we included a random factor for “quadrat nested within Pairs.” We used a Poisson distribution for the abundance of each species/genus and richness; a Beta distribution (logit link) for evenness, beta diversity, turnover and nestedness; and a Gamma distribution (log link) for inverse Simpson index.

Second, the variance ratio of the overall interspecific association of moss communities in each treatment was analyzed using the function *sp.assoc* from the R package *spaa* (Zhang et al., 2016). Spearman rank correlations of interspecies associations between all species pairs were calculated based on abundance data using the function *corr.test* from the R package *psych* (Revelle, 2022).

Third, we built a structural equation model (SEM) using the function *psem* from the R package *piecewiseSEM* (Lefcheck, 2016) to evaluate the direct and indirect effects of goose presence on beta diversity. The model was built based on prior knowledge (Herbivore impacts on plant diversity: Adler et al., 2001; Gauthier et al., 2004; Jasmin et al., 2008; Sjögersten et al., 2011; Relationship between alpha and beta diversity: Brocklehurst et al., 2018; Ricotta, 2017; Soininen et al., 2011). Justifications for each path in the initial SEM are summarized in Table S1–S9. In the model, we assumed that goose presence had the potential to alter beta diversity directly, as well as indirectly through changing richness, evenness, turnover, and nestedness. Additionally, the presence of geese was posited to affect turnover and nestedness directly, and indirectly through an effect on richness and evenness. To fit the SEM, we used the function *lme* with Pairs as a random effect for each model component, in order to test the relative contribution of treatment, species richness, evenness, turnover, and nestedness values to beta diversity at three spatial scales. However, the fit of the model was good only at the quadrat scale (a nonsignificant Fisher's *C* value with *p* > .05) and thus we report results at this scale only. We checked variance inflation for each model component to make sure that multicollinearity did not affect parameter estimates (variance inflation < 4). Species richness, evenness, beta diversity, turnover, and nestedness were natural log‐transformed to improve normality and homogeneity of variance.

Fourth, to test whether moss community composition responded to goose presence, we used nonmetric multidimensional scaling (NMDS) based on the Bray–Curtis dissimilarity measures of moss communities. Significance of the experimental treatment was assessed by applying permutational multivariate analysis of variance (PERMANOVA) on the Bray–Curtis dissimilarity measures (999 permutations). The NMDS was performed using the function *metaMDS* from R package *Vegan* (Oksanen et al., 2019). The data of moss community composition was natural log‐transformed to improve homogeneity of dispersion. Moss community composition between goose absence and presence still had different dispersion at the cell scale after transformation and thus we conducted the analysis only at the quadrat and exclosure scales.

Lastly, we tested the influence of treatment (fixed effect) on the relative abundance (proportion of one growth form relative to the total number of individuals) and richness of each moss growth form (pleurocarp and acrocarp) at the cell, quadrat, and exclosure scales using a generalized linear mixed model. We used a Poisson distribution for richness and a Beta distribution (logit link) for relative abundance. We included Pairs as a random variable and, at the cell scale, we also included a random factor for “quadrat nested within Pairs.” We applied post hoc Tukey's HSD test to determine differences in the relative abundance and richness between pleurocarp and acrocarp using the *emmeans* function from the ‘emmeans’ package.

## 3 RESULTS

### 3.1 Overall moss diversity, frequency, and abundance

A total of 52 moss species (16 pleurocarps, 34 acrocarps, and 2 *Sphagnum*) were present in our study area (all species are listed in Table S2). Among these, three typical fen species represented over 60% of total relative frequencies (ratio of the frequency of each species in all cells to the sum of all species frequencies): *Scorpidium* spp. (36.3%), *Bryum* spp. (15.6%, mainly *B. neodamense*), *Campylium stellatum* (9.3%). The species *Meesia* spp. (6.1%), *Sarmentypnum sarmentosum* (5.2%), and *Aulacomnium* spp. (5.0%) had a moderate relative frequency, whereas all other moss species had a relative frequency <5% (Table S3).

Among species with relative frequencies over 5.0%, goose presence increased the abundance of *Scorpidium* spp. (cell scale: *Z* = 115, *p* < .001; quadrat scale: *Z* = 88.2, *p* < .001; exclosure scale: *Z* = 88.3, *p* < .001), of *Bryum* spp. (cell scale: *Z* = 33.6; *p* < .001; quadrat scale: *Z* = 33.6, *p* < .001; exclosure: *Z* = 33.6, *p* < .001), and of *Sarmentypnum sarmentosum* (cell scale: *Z* = 5.50; *p* < .001, quadrat scale: *Z* = 39.4, *p* < .001, exclosure scale: *Z* = −39.4, *p* < .001), but reduced the abundance of *Aulacomnium* spp. (cell scale: *Z* = −25.0, *p* < .001; quadrat scale: *Z* = −25.0, *p* < .001; exclosure scale: *Z* = −25.0, *p* < .001) (Tables S4). The average density of moss shoots increased from 9.0 shoots cm^−2^ inside goose exclosures to 15.3 shoots cm^−2^ in presence of snow geese (*F* = 4.92, *p* = .04).

### 3.2 Plant diversity and interspecific association

**FIGURE 2:**
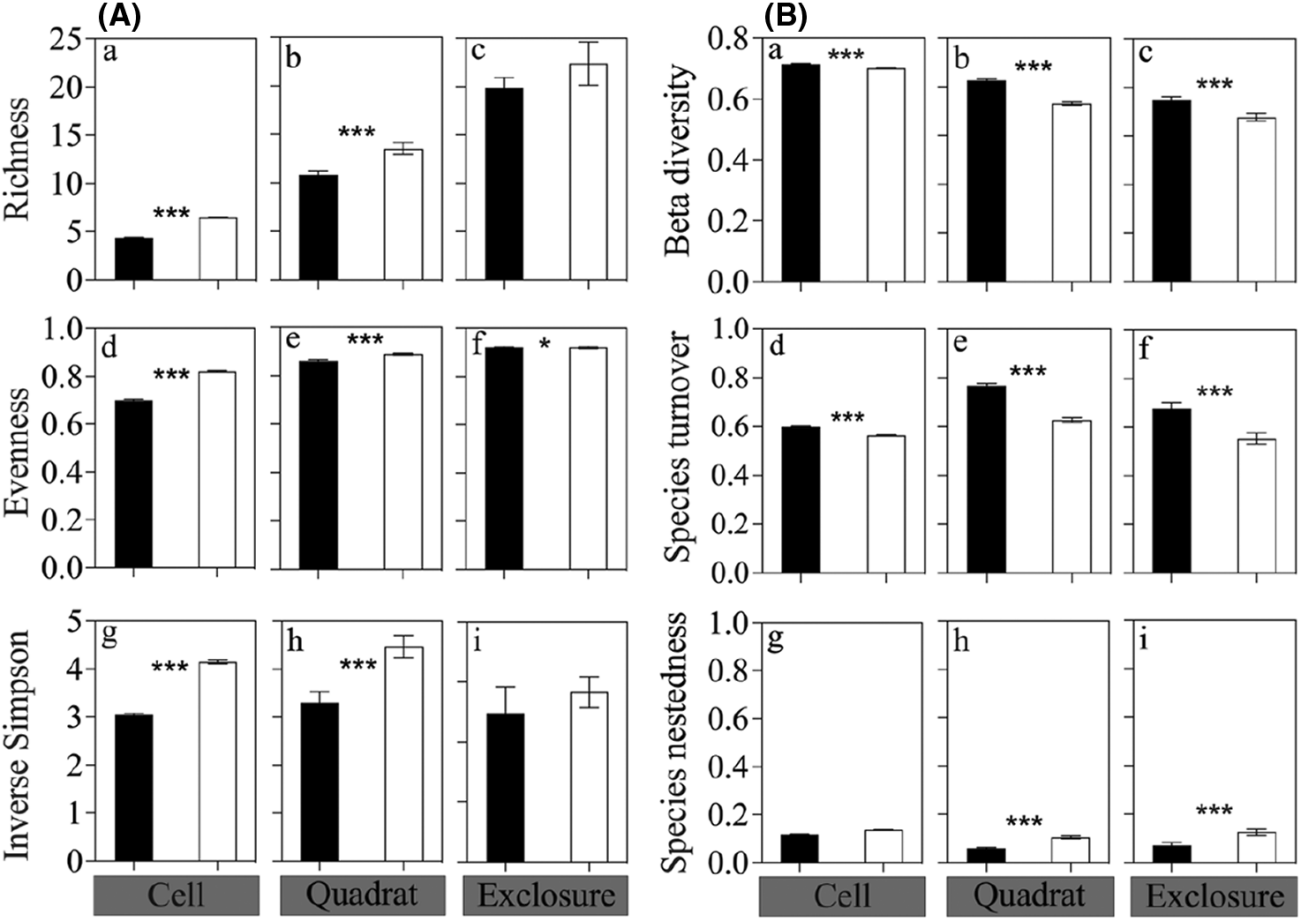
(A) Richness (number of species), evenness and inverse Simpson index, and (B) beta diversity, species turnover and species nestedness of moss communities in absence (black color) and presence (white color) of snow geese (inside and outside goose exclosures) at three spatial scales (Cell: 4 cm^2^, Quadrat: 100 cm^2^, Exclosure: 16 m^2^) on Bylot Island in the Canadian Arctic. Data are mean ± SE (Cell: *N* = 1250; Quadrat: *N* = 50; Exclosure: *N* = 10 in each treatment). Significant difference: *p < .05; ***p < .001.

Presence of snow geese significantly increased species richness, evenness, and inverse Simpson index at the cell (4 cm^2^, all *p* < .001) and quadrat scales (100 cm^2^, all *p* < .001), but not at the exclosure (16 m^2^) scale except for evenness (Figure 2A; Table S5).

Goose presence significantly decreased community dissimilarity (cell scale: *Z* = −25.5, *p* < .001; quadrat scale: *Z* = −11.7, *p* < .001; exclosure scale: *Z* = −6.1, *p* < .001) and species turnover at all spatial scales (cell scale: *Z* = −26.6, *p* < .001; quadrat scale: *Z* = −12.8, *p* < .001; exclosure scale: *Z* = −0.7, *p* < .001; Figure 2B; Table S5). Goose presence significantly increased species nestedness at the quadrat (*Z* = 8.6, *p* < .001) and exclosure scales (*Z* = 4.0, *p* = .007) but not at the cell scale. Species turnover was several times higher than species nestedness at the three spatial scales, indicating that community dissimilarity was mostly driven by species turnover (Figure 2B).

**FIGURE 3:**
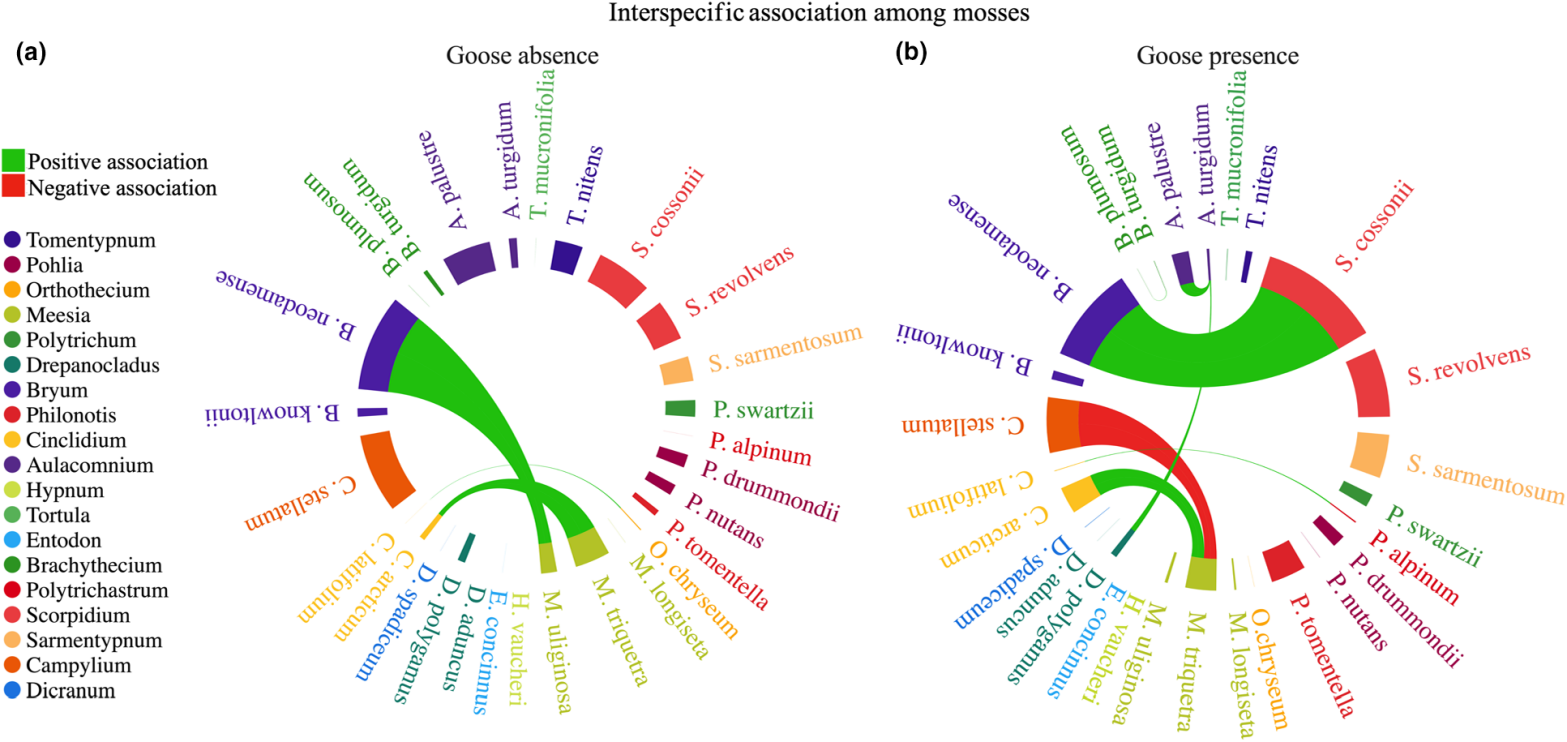
Illustration of the pairwise interspecific associations among mosses in absence (a) and presence (b) of snow geese inside and outside exclosure on Bylot Island in the Canadian Arctic. Edge width corresponds to the abundance of each specie. Note: interspecific associations between species with low abundance do not show clearly on the figure (see statistical results in Table S6). Each genus is represented by a different color.

In order to assess how goose presence affects the coexistence of moss species, we examined how the pairwise relationships between moss species responded to experimental exclusion. The variance ratios were larger than one in both absence (1.89, *W* = 94.61, *N* = 50, *p* < .05) and presence of snow geese (3.02, *W* = 150.91, *N* = 50, *p* < .05), indicating overall positive interspecific associations in moss communities of the two treatments. Positive associations (Spearman rank correlations ranging from .64 to 1.00) were observed in four species pairs in absence of snow geese whereas the remaining pairs showed neutral associations. In the presence of snow geese, six species pairs showed positive associations (Spearman rank correlation ranging from .60 to .82), one pair showed a negative association (Spearman rank correlation: −.68) and the remaining pairs showed neutral associations (Figure 3; Table S6).

### 3.3 Direct and indirect effects of snow geese on beta diversity

**FIGURE 4:**
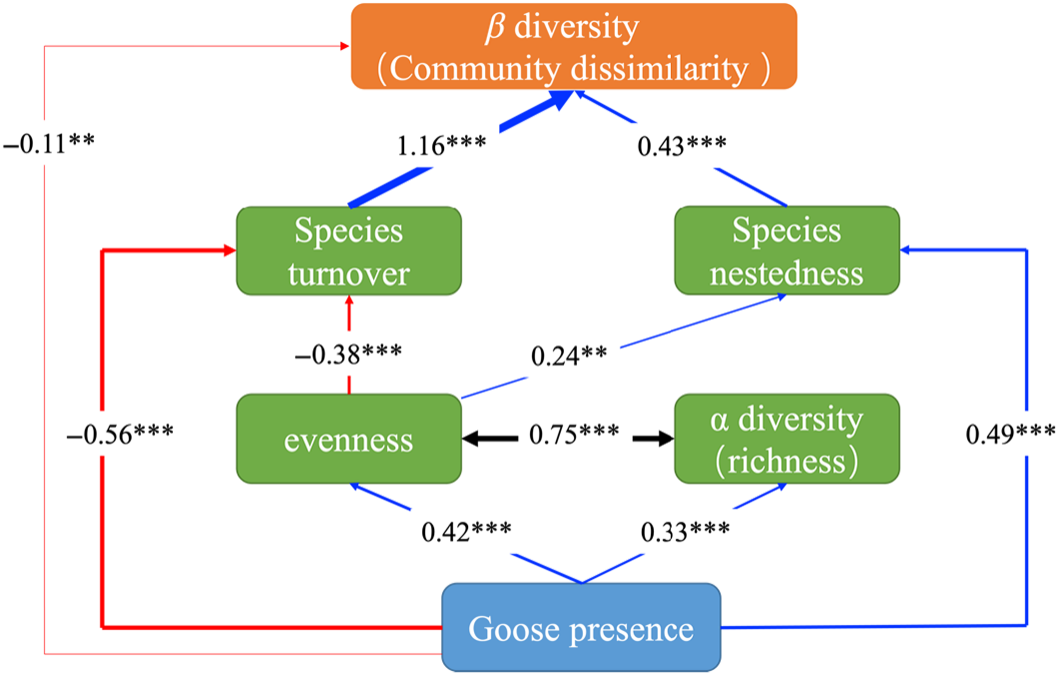
Results of the SEM showing direct and indirect effects of goose presence on beta diversity at the quadrat scale (100 cm^2^) on Bylot Island in the Canadian Arctic. The model fitted the data well (Fisher's *C* = 10.91, *p* = .091, df = 6). Boxes represent measured variables, and colored arrows represent relationships among variables. Numbers are standardized path coefficients. The width of the arrows indicates the strength of the path and line color represents positive (blue) and negative (red) relationships. Black arrows indicate correlations. Path significance: **p ≤ .01; ***p ≤ .001. Nonsignificant paths are not shown.

The structural equation model (SEM) clarified the direct and indirect effects of goose presence on the community dissimilarity (beta diversity) uncovered in the previous section at the quadrat scale. Community dissimilarity was strongly affected by species turnover, which was negatively affected by goose presence (Figure 4; Tables S7 and S8). The negative effect of snow geese on species turnover was also partly mediated by increased evenness, which negatively affected species turnover. On the other hand, goose presence positively affected species nestedness, which was also partly mediated by increased evenness.

### 3.4 Composition of moss communities

**FIGURE 5:**
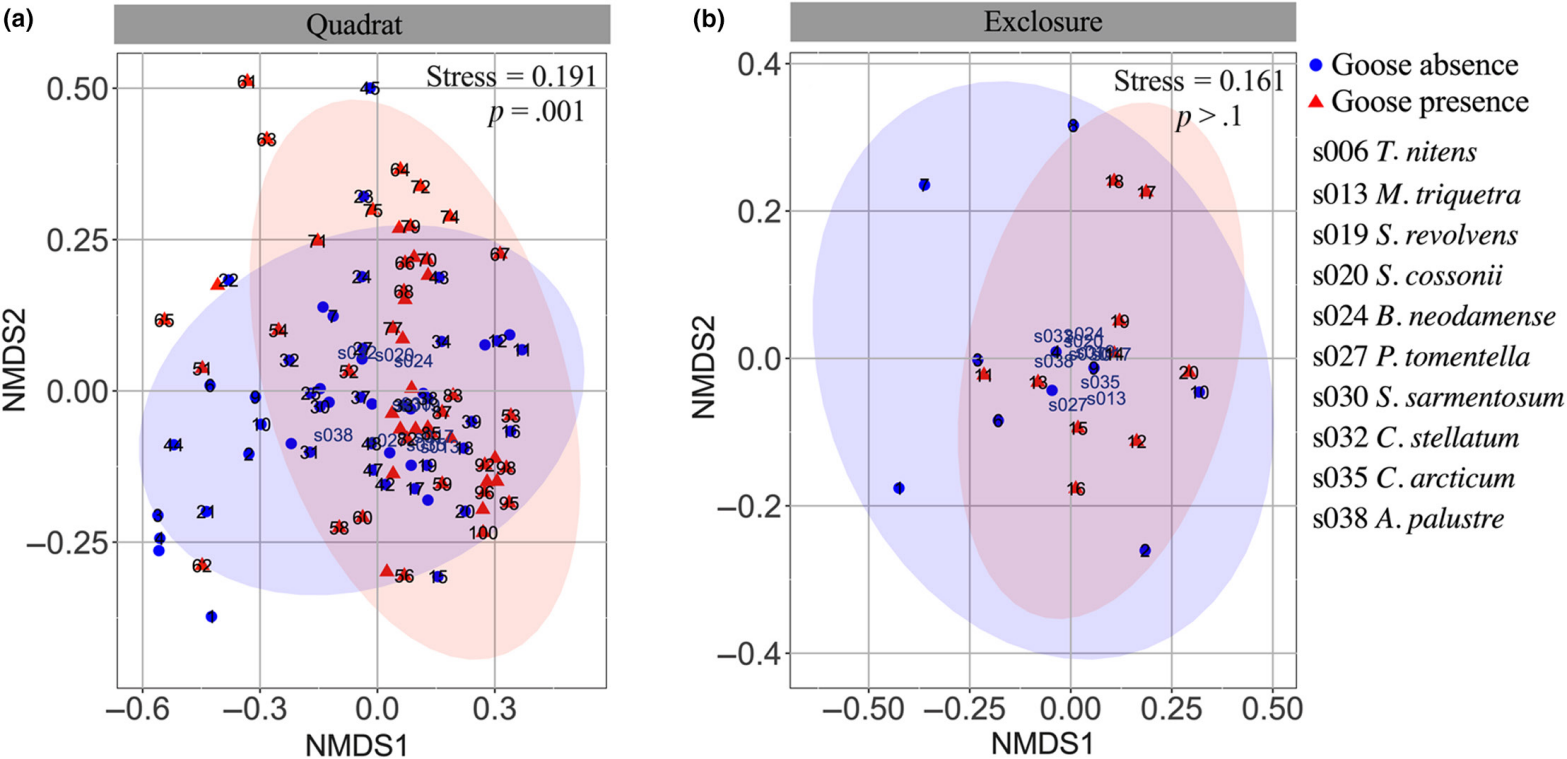
Plots of the first two axes of the nonmetric multidimensional scaling ordination analysis illustrating the moss community composition (measured as Bray–Curtis dissimilarities based on abundance data) in presence and absence of snow geese at quadrat (a) and exclosure (b) scales on Bylot Island in the Canadian Arctic. Blue numbers represent moss species (only the top 10 species are shown) and the black numbers represent the sampled sites. Stress represents the discrepancy between the original distances and the distances in the low‐dimensional space obtained by nonmetric multidimensional scaling.

Moss community composition at the quadrat scale differed in the presence and absence of snow geese along the first two axes of the NMDS analyses (*F* = 5.25, df = 1, *N* = 50, *p* = .001). However, at the exclosure scale, moss composition was similar in the absence and presence of snow geese (*F* = 1.51, df = 1, *N* = 10, *p* = .128) (Figure 5).

### 3.5 Species contribution to beta diversity

**FIGURE 6:**
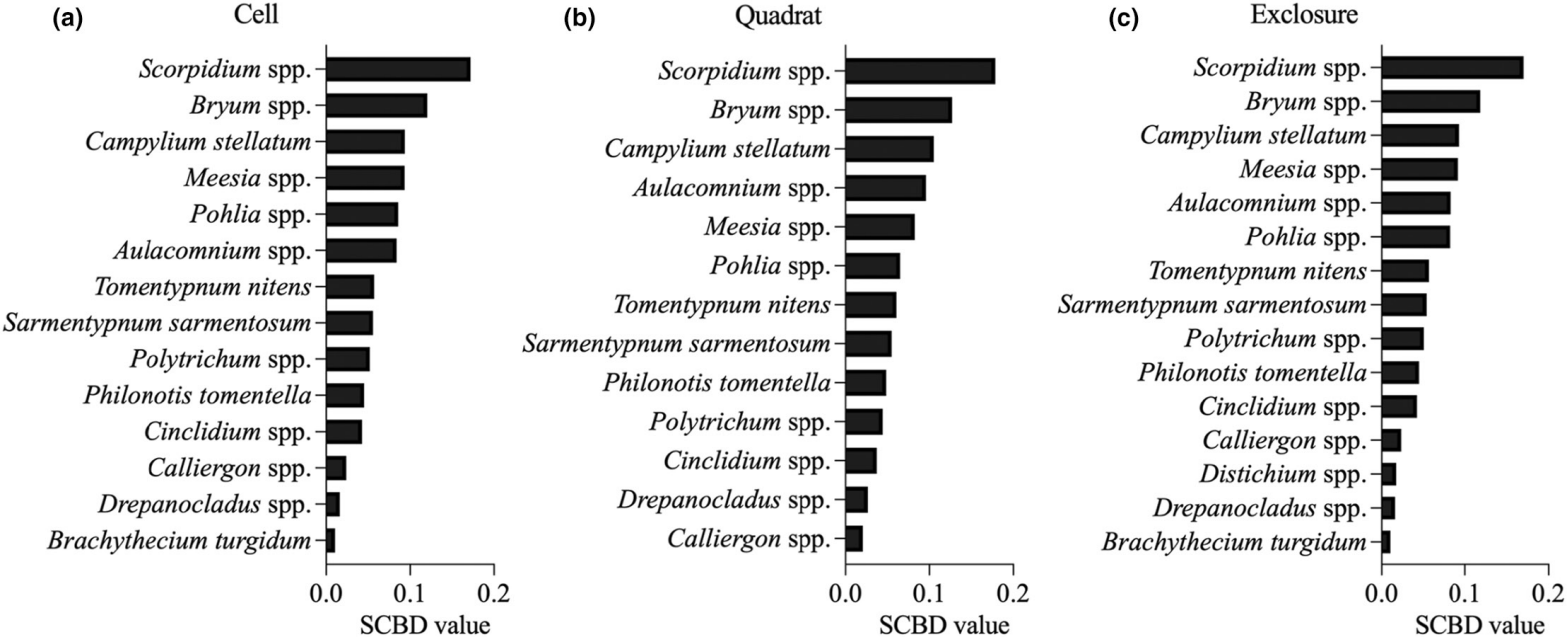
Species contribution to beta diversity (SCBD) values in moss communities at three spatial scales (Cell: 4 cm^2^, Quadrat: 100 cm^2^, Exclosure: 16 m^2^) on Bylot Island in the Canadian Arctic. Species with a value <.01 are not shown.

Species contribution to beta diversity (SCBD) ranges from 5.08 × 10^−7^ to 0.178. The top 10 species that contributed the most to beta diversity are the same at three scales and included *Scorpidium* spp., *Bryum* spp., *C. stellatum*, *Meesia* spp., *Aulacomnium* spp., *Pohlia* spp., *T. nitens*, *S. sarmentosum*, *P. tomentella*, and *Polytrichum* spp. (Figure 6).

### 3.6 Moss growth form

**FIGURE 7:**
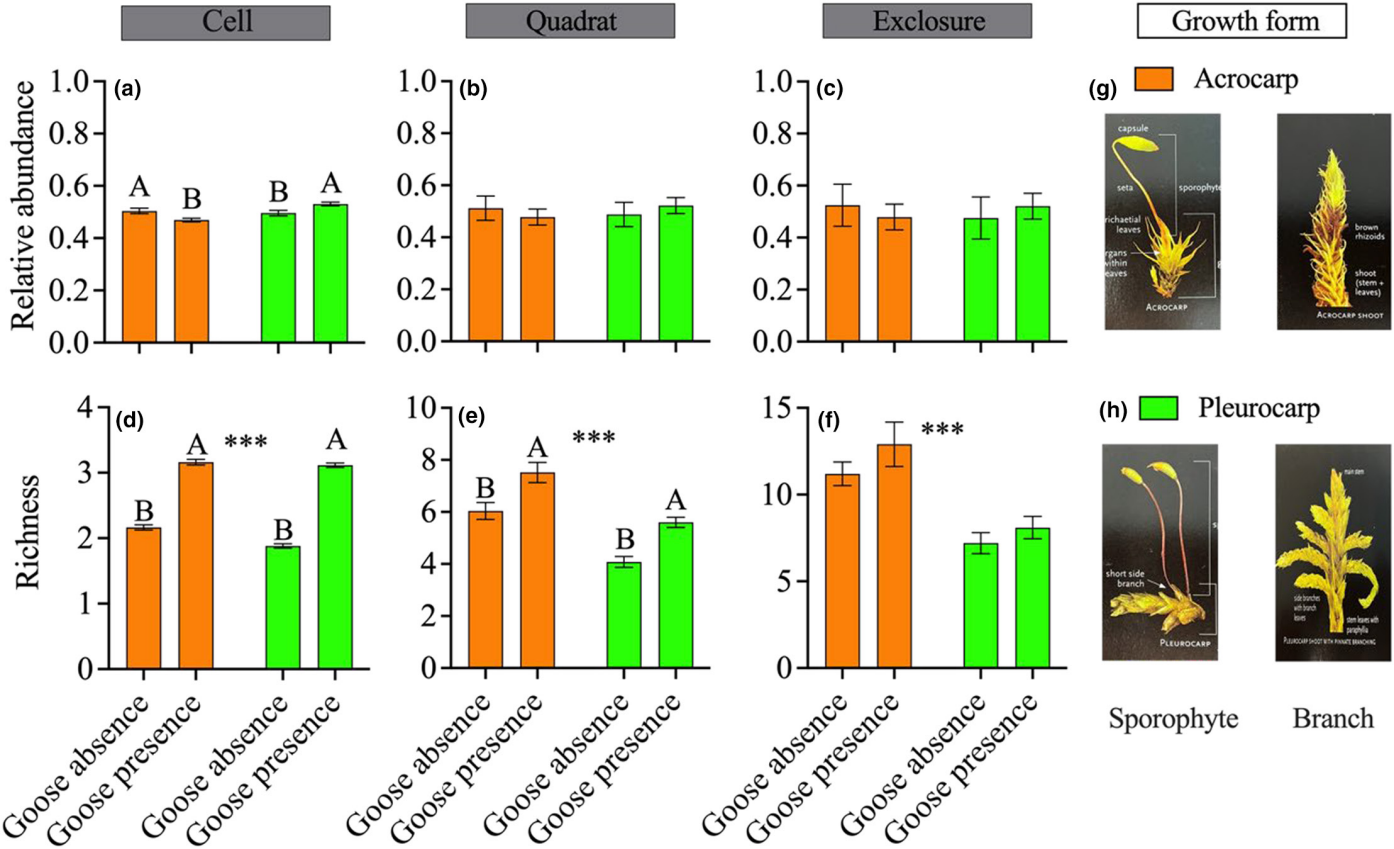
Relative abundance (proportion of one growth form relative to the total number of individuals) and richness (number of species) of acrocarpous and pleurocarpous moss species in absence and presence of snow geese at the three spatial scales (Cell: 4 cm^2^, Quadrat: 100 cm^2^, Exclosure: 16 m^2^; a–f) on Bylot Island in the Canadian Arctic. Data are mean ± SE (Cell: *N* = 1250; Quadrat: *N* = 50; Exclosure: *N* = 10 in each treatment). Uppercase letters (A, B) represent a significant difference between treatments and asterisks represent a significant difference between the two growth forms: ***p ≤ .001. Panels (g and h) illustrate the growth forms (Sporophyte and branch) of acrocarp and pleurocarp species (Jenkins, 2020).

Goose presence slightly increased the relative abundance of pleurocarpous species at the expanse aprocarpous ones (*Z* = 3.9, *p* < .001) at the cell scale but not at the quadrat and exclosure scales (Figure 7; Table S9). Goose presence also significantly increased the richness of acrocarpous (cell scale: *Z* = 13.0, *p* < .001; quadrat scale: *Z* = 2.9, *p* = .004) and pleurocarpous (cell scale: *Z* = 19.3, *p* < .001; quadrat scale: *Z* = 3.5, *p* < .001) species at the cell and quadrat scales. Additionally, the richness of acrocarpous species across both treatments was found to be higher than that of pleurocarpous species at the three spatial scales (cell scale: *F* = 17.64, *p* < .001; quadrat scale: *F* = 45.70, *p* < .001; exclosure scale: *F* = 42.73, *p* < .001), while there was no difference in overall relative abundance between the two growth forms at all spatial scales.

## 4 DISCUSSION

Using a unique, multiscale sampling and long‐term experiment, we evaluated the influence of a major herbivore of Arctic wetlands on moss community composition. Our results revealed the critical role of snow geese in promoting moss species coexistence and increasing homogeneity. Specifically, goose foraging altered moss community composition by increasing alpha diversity at small to moderate spatial scales (cell of 4 cm^2^ and quadrat of 100 cm^2^) and decreasing beta diversity at small to large spatial scales (cell of 4 cm^2^, quadrat of 100 cm^2^ and exclosure of 16 m^2^), as well as by increasing positive interactions between moss species pairs. Below, we discuss how these results relate to our initial hypotheses.

### 4.1 Snow geese and moss alpha diversity and coexistence

Whereas the impact of goose herbivory on the biomass, productivity, and species composition of vascular plants is by now rather well known (Gauthier et al., 2006), there is much less information regarding its role in regulating moss communities despite their ecological significance. We found that goose foraging increased alpha diversity (species richness, evenness and inverse Simpson index) at small and moderate scales, but not larger scale with the exception for evenness. Therefore, goose herbivory can promote moss species coexistence, but the effect is scale‐dependent. The likely explanation for this effect is that snow geese disturb the moss carpet when they grub for rhizomes, and possibly also when they trample the soil, opening regeneration niches for mosses (Gauthier et al., 2004; Jasmin et al., 2008). Several factors could explain the disappearance of the effect of goose presence on alpha diversity with upscaling. One could be the patchiness of goose grubbing at a small spatial scale, often due to snow melt patterns in spring (Speed et al., 2009), which may prevent rare species from reaching viable population sizes at large spatial scales. Alternatively, other external factors unrelated to snow geese (such as geomorphological conditions) may be more important in shaping community composition at a large spatial scale.

Goose foraging could also promote the coexistence of moss species by increasing positive pair interactions. Although interspecific associations within the moss community were generally positive both in absence and presence of snow geese, goose foraging increased positive associations between pairs of moss species. The facilitation was general among moss species and not limited to a few taxa. Moss associations can mutually promote each other's growth and survival by providing a suitable microclimate, ultimately increasing the diversity and overall health of the community (Callaway et al., 2002; During & Van Tooren, 1990; Rydin, 1993). This may increase the resilience of moss communities. At our site, snow geese reduce shading by consuming vascular plants, thus alleviating competition of mosses for light and likely promoting their richness. Moss shoot density was also 70% higher in presence than in absence of snow geese, which may have reduced risks of drying. Moreover, goose foraging changed the identity of species that interacted positively. For example, a positive interaction was detected between *B. neodamense* and *M. uliginosa* in absence of snow geese but between *B. neodamense* and *S. cossonii* in their presence, and the negative association between *C. stellatum* and *M. triquetra* only existed in presence of snow geese. Further experiments are needed to discover the mechanism(s) that cause changes in moss interspecific associations in presence of geese.

### 4.2 Snow geese, moss community composition change, and homogenization

We found that goose herbivory induced shifts in the composition of moss communities at small and moderate spatial scales. This is consistent with other studies showing an effect of herbivory on moss species in the Arctic (Oksanen & Moen, 1994; Virtanen, 2000). Snow geese probably influence species composition both directly and indirectly; for example, when they grub for rhizomes, they disturb the moss carpet by creating holes that may be colonized by various moss species (Jasmin et al., 2008). In addition, in the absence of grazing of vascular plants, litter accumulates (Deschamps et al., 2022), and competition for light probably limits the growth of moss species (an indirect effect). Thus, snow geese can act as a filter on plant community composition.

Our results show that herbivory can cause biotic homogenization (low beta diversity, i.e. loss of variation in plant composition) in moss communities by reducing species turnover at all spatial scales. Although goose foraging positively affected species nestedness, this effect was relatively weak compared to the negative effect on species turnover. The latter effect was partly mediated by an increase in species evenness due to goose foraging at moderate scale. This could be explained by the spreading of moss fragments by snow geese during grubbing, which can introduce new moss species to other areas and thus increases evenness. The top five species contributing to beta diversity are all abundant and dominant species at the study site. Previous work (e.g., Adler et al., 2001; Adler & Lauenroth, 2000; Rooney, 2009; Salgado‐Luarte et al., 2019) showed that homogeneous or some forms of selective grazing can both enhance homogenization. Our study adds to this evidence by showing that, with a controlled experiment, another herbivore feeding mode (grubbing) can have the same effect on Arctic moss communities. Although the spatial scales of our study are relatively small (≤16 m^2^) compared to some other studies (e.g. 1 m^2^ to 214 km^2^ in Liang et al., 2022; 4 m^2^ to 3 km^2^ in Beguin et al., 2022), they are considered large in the context of moss communities, whose fine‐scale is measured in centimeters (Jasmin et al., 2008). The impact of herbivory on beta diversity of plants, excluding tree species, is also usually found at relatively small spatial scales. For instance, in a similar study, the beta diversity of vascular plants was reduced by deer herbivory at a 4 m^2^ scale, but the effect disappeared with upscaling (Beguin et al., 2022).

### 4.3 Snow geese and moss growth forms

We expected that pleurocarp growth forms would be favored over acrocarp growth forms in areas exposed to goose foraging due to their functional traits, but this was only confirmed at small spatial scale and the effect was small for relative abundance. This result nonetheless highlights the high regeneration abilities of pleurocarp species (Glime, 2017; Li & Vitt, 1994). Other studies showed that pleurocarpous mosses could establish and survive well on disturbed sites (Lesica et al., 1991; Márialigeti et al., 2009). However, it is worth noting that there is much variability among different moss species, and some acrocarpous species may be able to grow and regenerate quite quickly. In contrast, some pleurocarpous species may be slow‐growing and less apt at colonizing new habitats. For instance, *Tomentypnum nitens*, a pleurocarpous species, was negatively affected by goose foraging, while some acrocarpous species like *Cinclidium* spp. and *Philonotis tomentella* were favored by goose foraging (Table S4).

### 4.4 Goose herbivory and top‐down ecological regulation

The top‐down regulation hypothesis predicts that herbivory reduces plant abundance, biomass, and survival but increases diversity through the disproportionate consumption of dominant species, which inhibits competitive exclusion (Jia et al., 2018). In Arctic tundra wetlands, mosses form a major component of plant diversity (Turetsky et al., 2012). However, some studies suggest that mosses are under threat in Arctic regions due to rapid climate warming, with major feedback consequences for ecosystem function (Cornelissen et al., 2007; Deane‐Coe & Stanton, 2017; Lang et al., 2011; Street et al., 2012). Indeed, vascular plants like graminoids should become more abundant in northern wetlands because of global warming, which will increase competition for light for the moss layer due to the shading created by vascular plants (Bao et al., 2022; Gignac et al., 2022; Le et al., 2022). However, snow geese can consume up to 60% of the annual production of wetland graminoids in the High Arctic (Gauthier et al., 1995; Valéry et al., 2010). Their grazing can thus alter the competitive balance between mosses and vascular plants by removing graminoid biomass, preventing litter accumulation, and changing the soil thermal profile and hydrology, which indirectly promotes high moss biomass (Deschamps et al., 2022; Gauthier et al., 2004). Our study adds to these effects of goose foraging as we found that goose presence increases alpha diversity and promotes moss coexistence. Taken together, these results show that goose herbivory is an essential biotic agent releasing mosses from competitive exclusion by vascular plants. Such top‐down regulation can delay some of the expected changes in plant communities of Arctic wetlands, especially moss communities, in response to climate warming (Deschamps et al., 2022).

## 5 CONCLUSIONS

Goose foraging changes the composition of moss communities by increasing alpha diversity (H1) and reducing beta diversity (H3), as well as by increasing positive interactions between moss species pairs (H2), thereby supporting our initial hypotheses. Pleurocarps are somewhat favored over acrocarps in areas exposed to goose foraging due to their functional traits (H4) but only at a small spatial scale, which offers only partial support for our initial hypothesis. However, the effect of goose grazing on alpha diversity disappears with upscaling, possibly because other factors (e.g. patchiness of goose grubbing and geomorphological conditions) become more important at large spatial scales. Our study shows the critical role of goose herbivory in promoting the coexistence of moss species and increasing homogeneity. At intermediate levels of herbivory, goose foraging is a driver of spatial patterns in plant communities and may play an important role in the conservation of biodiversity in Arctic tundra wetlands in a context of global change.

## AUTHOR CONTRIBUTIONS

**Chao Liu:** Data curation (equal); investigation (equal); writing – original draft (equal); writing – review and editing (equal). **Gilles Gauthier:** Funding acquisition (equal); methodology (equal); supervision (equal); writing – review and editing (equal). **Charles Gignac:** Data curation (equal); investigation (equal); writing – review and editing (equal). **Esther Lévesque:** Funding acquisition (equal); methodology (equal); supervision (equal); writing – review and editing (equal). **Line Rochefort:** Data curation (equal); funding acquisition (equal); methodology (equal); supervision (equal); writing – review and editing (equal).

## FUNDING INFORMATION

Financial support for this study was provided by the Natural Sciences and Engineering Research Council of Canada, the ArcticNet Network of Centre of Excellence, and the Northern Scientific Training Program of Polar Knowledge Canada. We also received logistical support from the Polar Continental Shelf Program (PCSP) of Natural Resources Canada and the Centre d'études Nordiques.

## CONFLICT OF INTEREST STATEMENT

The authors have no conflict of interest.

## Supporting information


**Tables S1–S9**.

## Data Availability

Data used for this analysis and R code of all analyses are available from figshare Digital Repository: https://doi.org/10.6084/m9.figshare.24134883.v2.
