# Supplementary material for: Scale‐dependent effects of herbivory on moss communities in Arctic wetlands: A 25‐year experiment
Source: Ecol Evol. 2024 Apr 25;14(4):e11272. doi: 10.1002/ece3.11272 (PMC11043830; doi:10.1002/ece3.11272)
Supplement: Supplementary file 1 — Tables S1–S9. [file ECE3-14-e11272-s001.docx]

**Tables**

Table S1. Justifications of each pathway included in the initial structural equation model (SEM) to test the direct and indirect effects of goose presence on the diversity of moss communities.

| **Pathways** | **Hypotheses and mechanisms** | **References** |
| --- | --- | --- |
| Goose presence -> species richness | Goose foraging promotes moss species richness by opening regeneration niches and reducing living vascular plant biomass. | (﻿Gauthier et al. 2004; Jasmin et al. 2008; ﻿Sjögersten et al. 2011). |
| Goose presence -> evenness | Goose foraging contributes to the spreading of individual ﻿moss species, which increases their dominance, and thus evenness. | (Jasmin et al. 2008) |
| Goose presence -> beta diversity, species turnover and species nestedness^1^ | Herbivores can decrease or increase beta diversity because their effects on vegetation heterogeneity depend on their grazing pattern. Specifically, grazing in patches enhances vegetation heterogeneity, whereas homogeneous or selective grazing reduces it. | (Adler et al. 2001) |
| Evenness-> beta diversity, species turnover and species nestedness | A more even distribution of plant abundance results in higher beta diversity estimates ﻿because the variation in mean pairwise distances is strongly influenced by the structure of the distribution of plant abundance. | (Ricotta, 2016; Brocklehurst et al.2018) |
| Species richness<-> Evenness (correlation) | Although correlations between species richness and evenness can range from negative to positive, positive correlations are more frequent. | (Soininen et al. 2012) |

^1^ Species turnover and species nestedness are components of beta diversity (Baselga, 2017)

Table S2. Mosses species found in the study area.

Table S3. Relative moss species frequency (expressed as the ratio of the frequency of each species in all samples, N = 2500, to the sum of all species frequencies) in our sampled plots presented in decreasing order.

| Species name | Frequency (%) |
| --- | --- |
| *Scorpidium* spp. | 36.3 |
| *Bryum* spp. (mainly *B. neodamense*) | 15.6 |
| *Campylium stellatum* | 9.3 |
| *Meesia* spp. | 6.1 |
| *Sarmentypnum sarmentosum* | 5.2 |
| *Aulacomnium* spp. | 5.0 |
| *Polytrichum* spp. | 4.7 |
| *Philonotis tomentella* | 3.4 |
| *Cinclidium* spp. (mainly *C. arcticum*) | 2.8 |
| *Pohlia* spp. | 2.5 |
| *Tomentypnum nitens* | 2.1 |
| *Calliergon* spp. | 2.1 |
| *Drepanocladus* spp. | 2.0 |
| The other 20 species | < 0.5 |

Table S4. Statistical results from generalized linear mixed models analyzing the influence of goose presence (fixed effect, *df* = 1) on the abundance of moss species (relative moss species frequency larger than 2.0 %) at the three spatial scales (Cell: N = 1250; Quadrat: N = 50; Exclosure: N = 10 in each treatment) on Bylot Island in the Canadian Arctic. Slope estimates are provided with SE in parentheses and statistical significance is highlighted in bold. We report the marginal R^2^ accounting for variance explained by fixed factors only. RMSE, ﻿root mean square error.

| Species | Cell (4 cm^2^) | | | Quadrat (100 cm^2^) | | | Exclosure (16 m^2^) | | |
| --- | --- | --- | --- | --- | --- | --- | --- | --- | --- |
| *Shoots gained*^1^ |  | | |  | | |  | | |
| *Bryum* spp. | **Estimate**: 0.445 (0.01)  ***Z***: 33.6  ***P***: **<0.001**  **R^2^**_:_ 0.019  **RMSE**: 13.41 | | | **Estimate**: 0.445 (0.01)  ***Z***: 33.6  ***P***: **<0.001**  **R^2^**_:_ 0.045  **RMSE**: 253.1 | | | **Estimate**: 0.445 (0.01)  ***Z***: 33.6  ***P***: **<0.001**  **R^2^**_:_ 0.047  **RMSE**: 823.1 | | |
| *Calliergon* spp*.* | **Estimate**: 1.713 (0.05)  ***Z***: 34.3  ***P***: **<0.001**  **R^2^**_:_ 0.063  **RMSE**: 3.279 | | | **Estimate**: 1.675 (0.06)  ***Z***: 26.7  ***P***: **<0.001**  **R^2^**_:_ 0.127  **RMSE**: 38.67 | | | **Estimate**: 1.675 (0.06)  ***Z***: 26.7  ***P***: **<0.001**  **R^2^**_:_ 0.135  **RMSE**: 63.33 | | |
| *Campylium stellatum* | **Estimate**: 0.091 (0.02)  ***Z***: 5.32  ***P***: **<0.001**  **R^2^**_:_ 0.000  **RMSE**: 7.122 | | | **Estimate**: 0.091 (0.02)  ***Z***: 5.32  ***P***: **<0.001**  **R^2^**_:_ 0.000  **RMSE**:138.7 | | | **Estimate**: 0.091 (0.02)  ***Z***: 5.32  ***P***: **<0.001**  **R^2^**_:_ 0.000  **RMSE**:306.6 | | |
| *Cinclidium* spp. | **Estimate**: 2.028 (0.05)  ***Z***: 41.6  ***P***: **<0.001**  **R^2^**_:_ 0.147  **RMSE**: 3.085 | | | **Estimate**: 2.028 (0.05)  ***Z***: 41.6  ***P***: **<0.001**  **R^2^**_:_ 0.295  **RMSE**: 73.71 | | | **Estimate**: 2.028 (0.05)  ***Z***: 41.6  ***P***: **<0.001**  **R^2^**_:_ 0.308  **RMSE**: 88.45 | | |
| *Meesia* spp. | **Estimate**: 0.088 (0.02)  ***Z***: 4.14  ***P***: **<0.001**  **R^2^**_:_ 0.000  **RMSE**: 7.646 | | | **Estimate**: 0.084 (0.02)  ***Z***: 3.97  ***P***: **<0.001**  **R^2^**_:_ 0.000  **RMSE**: 167.1 | | | **Estimate**: 0.084 (0.02)  ***Z***: 3.97  ***P***: **<0.001**  **R^2^**_:_ 0.000  **RMSE**: 249.3 | | |
| *Philonotis tomentella* | **Estimate**: 2.153 (0.05)  ***Z***: 46.9  ***P***: **<0.001**  **R^2^**_:_ 0.097  **RMSE**: 3.681 | | | **Estimate**: 2.153 (0.05)  ***Z***: 46.9  ***P***: **<0.001**  **R^2^**_:_ 0.138  **RMSE**: 82.83 | | | **Estimate**: 2.153 (0.05)  ***Z***: 46.9  ***P***: **<0.001**  **R^2^**_:_ 0.144  **RMSE**: 74.35 | | |
| *Polytrichum* spp. | **Estimate**: 0.269 (0.02)  ***Z***: 11.1  ***P***: **<0.001**  **R^2^**_:_ 0.002  **RMSE**: 5.612 | | | **Estimate**: 0.303 (0.03)  ***Z***: 9.3  ***P***: **<0.001**  **R^2^**_:_ 0.018  **RMSE**: 53.66 | | | **Estimate**: 0.303 (0.03)  ***Z***: 9.3  ***P***: **<0.001**  **R^2^**_:_ 0.019  **RMSE**: 141.0 | | |
| *Sarmentypnum sarmentosum* | **Estimate**: 0.288 (0.05)  ***Z***: 5.50  ***P***: **<0.001**  **R^2^**_:_ 0.002  **RMSE**: 1.860 | | | **Estimate**: 1.014 (0.03)  ***Z***: 39.4  ***P***: **<0.001**  **R^2^**_:_ 0.116  **RMSE**: 92.57 | | | **Estimate**: 1.014 (0.03)  ***Z***: 39.4  ***P***: **<0.001**  **R^2^**_:_ 0.121  **RMSE**: 104.2 | | |
| *Scorpidium* spp. | **Estimate**: 1.168 (0.01)  ***Z***: 115  ***P***: **<0.001**  **R^2^**_:_ 0.154  **RMSE**: 29.17 | | | **Estimate**: 1.130 (0.01)  ***Z***: 88.2  ***P***: **<0.001**  **R^2^**_:_ 0.282  **RMSE**: 301.6 | | | **Estimate**: 1.130 (0.01)  ***Z***: 88.3  ***P***: **<0.001**  **R^2^**_:_ 0.292  **RMSE**:917.0 | | |
| *Shoots lost*^2^ |  |  |  |  |  |  |  |  |  |
| *Aulacomnium* spp. | **Estimate**: -0.612 (0.02)  ***Z***: **-**25.0  ***P***: **<0.001**  **R^2^**_:_ 0.020  **RMSE**: 5.156 | | | **Estimate**: -0.612 (0.02)  ***Z***: **-**25.0  ***P***: **<0.001**  **R^2^_GLMM_**_:_ 0.062  **RMSE**: 106.4 | | | **Estimate**: -0.612 (0.02)  ***Z***: **-**25.0  ***P***: **<0.001**  **R^2^_GLMM_**_:_ 0.065  **RMSE**: 172.7 | | |
| *Pohlia* spp. | **Estimate**: -0.158 (0.00)  ***Z***: **-**321.6  ***P***: **<0.001**  **R^2^**_:_ 0.000  **RMSE**: 4.891 | | | **Estimate**: -0.158 (0.03)  ***Z***: **-**4.81  ***P***: **<0.001**  **R^2^**_:_ 0.000  **RMSE**: 45.78 | | | **Estimate**: -0.158 (0.03)  ***Z***: **-**4.81  ***P***: **<0.001**  **R^2^**_:_ 0.000  **RMSE**: 155.3 | | |
| *Tomentypnum nitens* | **Estimate**: -0.994 (0.04)  ***Z***: **-**24.8  ***P***: **<0.001**  **R^2^**_:_ 0.017  **RMSE**: 3.011 | | | **Estimate**: -0.994 (0.04)  ***Z***: **-**24.8  ***P***: **<0.001**  **R^2^**_:_ 0.038  **RMSE**: 76.7 | | | **Estimate**: -0.994 (0.04)  ***Z***: **-**24.8  ***P***: **<0.001**  **R^2^**_:_ 0.040  **RMSE**: 153.0 | | |

^1^ Shoots gained means that abundance of the species increased in presence of snow geese.

^2^ Shoots lost means that abundance of the species decreased in presence of snow geese.

Table S5. Statistical results from generalized linear mixed models analyzing the influence of goose presence (fixed effect, *df* =1) on plant diversity measures of moss communities at the three spatial scales on Bylot Island in the Canadian Arctic. Slope estimates are provided with SE in parentheses and statistical significance is highlighted in bold. We report the marginal R^2^ accounting for variance explained by fixed factors only. RMSE, ﻿root mean square error.

| Response variable | Cell (4 cm^2^) | | Quadrat (100 cm^2^) | Exclosure (16 m^2^) |
| --- | --- | --- | --- | --- |
| Alpha diversity (species richness) | | **Estimate**: 0.413 (0.02)  ***Z***: 23.4  ***P***: **<0.001**  **R^2^**_:_ 0.156  **RMSE**: 1.463 | **Estimate**: 0.222 (0.06)  ***Z***: 3.87  ***P***: **<0.001**  **R^2^**_:_ 0.091  **RMSE**: 2.397 | **Estimate**: 0.118 (0.10)  ***Z***: 1.22  ***P***: 0.224  **R^2^**_:_ 0.047  **RMSE**: 2.933 |
| Evenness | | **Estimate**: 0.525 (0.03)  ***Z***: 18.6  ***P***: **<0.001**  **R^2^**_:_ 0.540  **RMSE**: 0.126 | **Estimate**: 0.270 (0.04)  ***Z***: 6.4  ***P***: **<0.001**  **R^2^**_:_ 0.327  **RMSE**: 0.023 | **Estimate**: 0.082 (0.04)  ***Z***: 2.06  ***P***: **0.039**  **R^2^**_:_ 0.052  **RMSE**: 0.005 |
| Simpson index | | **Estimate**: 0.305 (0.01)  ***t***: 26.5  ***P***: **<0.001**  **R^2^**_:_ 0.200  **RMSE**: 0.104 | **Estimate**: 0.334 (0.07)  ***t***: 4.99  ***P***: **<0.001**  **R^2^**_:_ 0.151  **RMSE**: 1.262 | **Estimate**: 0.202 (0.12)  ***t***: 1.62  ***P***: 0.105  **R^2^**_:_ 0.057  **RMSE**: 1.582 |
| Beta diversity (community dissimilarity) | | **Estimate**: -0.325 (0.01)  ***Z***: -25.5  ***P***: **<0.001**  **R^2^**_:_ 0.386  **RMSE**: 0.038 | **Estimate**: -0.565 (0.05)  ***Z***: -11.7  ***P***: **<0.001**  **R^2^**_:_ 0.856  **RMSE**: 0.037 | **Estimate**: -0.348 (0.06)  ***Z***: -6.06  ***P***: **<0.001**  **R^2^**_:_ 0.452  **RMSE**: 0.020 |
| Species turnover | | **Estimate**: -0.379 (0.01)  ***Z***: -26.6  ***P***: **<0.001**  **R^2^**_:_ 0.375  **RMSE**: 0.059 | **Estimate**: -0.683 (0.05)  ***Z***: -12.8  ***P***: **<0.001**  **R^2^**_:_ 0.826  **RMSE**: 0.052 | **Estimate**: -0.540 (0.08)  ***Z***: -0.70  ***P***: **<0.001**  **R^2^**_:_ 0.570  **RMSE**: 0.031 |
| Species nestedness | | **Estimate**: 0.003 (0.02)  ***Z***: 0.12  ***P***: 0.901  **R^2^**_:_ 0.000  **RMSE**: 0.071 | **Estimate**: 0.633 (0.07)  ***Z***: 8.63  ***P***: **<0.001**  **R^2^**_:_ 0.397  **RMSE**: 0.028 | **Estimate**: 0.641 (0.16)  ***Z***: 3.95  ***P***: **<0.001**  **R^2^**_:_ 0.434  **RMSE**: 0.029 |

Table S6. Spearman rank correlations of moss species pairs showing significant associations (all *P* ≤ 0.001, N = 50) in absence and presence of snow geese on Bylot Island in the Canadian Arctic.

| Treatment | Species pairs | | Association | Spearman rank correlation |
| --- | --- | --- | --- | --- |
| Goose absence | | *Bryum neodamense—Meesia uliginosa* | Positive | **0.64** |
|  |  | *Cinclidium latifolium—Meesia triquetra* | Positive | **0.73** |
|  |  | *Entodon concinnus—Tortula mucronifolia* | Positive | **1.00** |
|  |  | *Cinclidium latifolium—Orthothecium chryseum* | Positive | **1.00** |
| Goose presence | | *Campylium stellatum—Meesia triquetra* | Negative | **-0.68** |
|  |  | *Cinclidium arcticum—Meesia triquetra* | Positive | **0.66** |
|  |  | *Cinclidium latifolium—Polytrichum alpinum* | Positive | **0.69** |
|  |  | *Bryum neodamense—Scorpidium cossonii* | Positive | **0.60** |
|  |  | *Aulacomnium turgidum—Drepanocladus polyganum* | Positive | **0.63** |
|  |  | *Aulacomnium turgidum—Aulacomnium palustre* | Positive | **0.64** |
|  |  | *Brachythecium plumosum—Brachythecium turgidum* | Positive | **0.82** |

Table S7. Standardized coefficients (unstandardized for the correlation) and significance level of the different paths of our final SEM (N = 100) examining direct and indirect effects of goose presence on beta diversity at the quadrat scale on Bylot Island in the Canadian Arctic (see Fig. 4). All variables were natural log-transformed to compare effect sizes. Significant effects (*P* ≤ 0.05) are highlighted in bold.

| Pathways | Estimate | Std.Error | *df* | *t*-value | *P*-value | Std.Estimate |
| --- | --- | --- | --- | --- | --- | --- |
| Goose presence -> Beta diversity | -0.019 | 0.006 | 86 | -2.98 | **0.004** | -0.110 |
| Species turnover-> Beta diversity | 0.683 | 0.036 | 86 | 19.07 | **<0.001** | 1.164 |
| Species nestedness-> Beta diversity | 0.071 | 0.008 | 86 | 8.76 | **<0.001** | 0.428 |
| Goose presence -> Species turnover | -0.162 | 0.018 | 88 | -9.11 | **<0.001** | -0.558 |
| Evenness->Species turnover | -1.275 | 0.257 | 88 | -4.96 | **<0.001** | -0.377 |
| Goose presence ->Species nestedness | 0.508 | 0.078 | 88 | 6.54 | **<0.001** | 0.494 |
| Evenness->Species nestedness | 2.836 | 1.128 | 88 | 2.51 | **0.014** | 0.237 |
| Goose presence ->Evenness | 0.036 | 0.006 | 89 | 6.11 | **<0.001** | 0.416 |
| Goose presence ->Species richness | 0.216 | 0.044 | 89 | 4.96 | **<0.001** | 0.329 |
| Correlation | Estimate | Std.Error | *df* | *t*-value | *P*-value | Std.Estimate |
| Species richness <->Evenness | 0.749 | - | 99 | 11.12 | **<0.001** | 0.749 |

Table S8. Variance of endogenous variables (i.e. the response variable in each component model) explained in the structural equation models of Fig. 4. For all response variables we used a Gaussian distribution and an identity link.

| Variable | R^2^m | R^2^c |
| --- | --- | --- |
| Beta diversity | 0.93 | 0.94 |
| Species turnover | 0.60 | 0.74 |
| Species nestedness | 0.37 | 0.62 |
| Richness | 0.10 | 0.59 |
| Evenness | 0.17 | 0.56 |

R^2^m, model R-squared, represents the proportion of variance in the endogenous variable that is explained by the entire structural equation model.

R^2^c, construct-specific R-squared, represents the proportion of variance in the endogenous variable that is explained by its corresponding latent construct in the model.

Table S9. Statistical results from generalized linear mixed models analyzing the influence of goose presence (fixed effect, *df* =1) on the relative abundance of pleurocarp vs aprocarp moss species, and richness of acrocarp and pleurocarp moss species at three spatial scales on Bylot Island in the Canadian Arctic. Statistical significance is highlighted in bold. We report the marginal R^2^ accounting for variance explained by fixed factors only. RMSE, ﻿root mean square error.

| Response variable | | Cell (4 cm^2^) | Quadrat (100 cm^2^) | Exclosure (16 m^2^) |
| --- | --- | --- | --- | --- |
| Relative abundance of pleurocarp | | **Estimate**: 0.190 (0.05)  ***Z***: 3.85  ***P***: **<0.001**  **R^2^**_:_ 0.010  **RMSE**: 0.252 | **Estimate**: 0.203 (0.20)  ***Z***: 0.10  ***P***: 0.319  **R^2^**_:_ 0.057  **RMSE**: 0.246 | **Estimate**: 0.189 (0.34)  ***Z***: 0.56  ***P***: 0.574  **R^2^**_:_ 0.111  **RMSE**: 0.177 |
| Richness | Acrocarp | **Estimate**: 0.274 (0.02)  ***Z***: 13.0  ***P***: **<0.001**  **R^2^**_:_ 0.055  **RMSE**: 1.135 | **Estimate**: 0.222 (0.08)  ***Z***: 2.88  ***P***: **0.004**  **R^2^**_:_ 0.065  **RMSE**: 1.984 | **Estimate**: 0.141 (0.13)  ***Z***: 1.09  ***P***: 0.274  **R^2^**_:_ 0.056  **RMSE**: 2.723 |
|  | Pleurocarp | **Estimate**: 0.504 (0.03)  ***Z***: 19.3  ***P***: **<0.001**  **R^2^**_:_ 0.124  **RMSE**: 0.899 | **Estimate**: 0.322 (0.09)  ***Z***: 3.50  ***P***: **<0.001**  **R^2^**_:_ 0.113  **RMSE**: 1.168 | **Estimate**: 0.118 (0.16)  ***Z***: 0.73  ***P***: 0.467  **R^2^**_:_ 0.029  **RMSE**: 1.877 |
